# Nanotechnology in Wound Healing; Semisolid Dosage Forms Containing Curcumin-Ampicillin Solid Lipid Nanoparticles, *in-Vitro*, *Ex-Vivo* and *in-Vivo* Characteristics

**DOI:** 10.15171/apb.2018.046

**Published:** 2018-08-29

**Authors:** Solmaz Ghaffari, Faezeh Alihosseini, Seyed Mahdi Rezayat Sorkhabadi, Sepideh Arbabi Bidgoli, Seyyedeh Elaheh Mousavi, Setareh Haghighat, Ahoo Afshar Nasab, Nooshin Kianvash

**Affiliations:** ^1^Pharmaceutical Sciences Research Center, Pharmaceutical Sciences Branch, Islamic Azad University (IAUPS), Tehran, Iran.; ^2^Young Researchers and Elite Club, Pharmaceutical Sciences Branch, Islamic Azad University (IAUPS), Tehran, Iran.; ^3^Department of Medical Nanotechnology, School of Advanced Sciences and Technology in Medicine, Tehran University of Medical Sciences (TUMS), Tehran, Iran.; ^4^Department of Pharmacology and Toxicology, Pharmaceutical Sciences Branch, Islamic Azad University, (IAUPS), Tehran, Iran.; ^5^Department of Toxicology, Pharmaceutical Sciences Branch, Islamic Azad University (IAUPS), Tehran, Iran.; ^6^Department of Pharmacology, School of Medicine, Tehran University of Medical Sciences, Tehran, Iran.; ^7^Department of Microbiology, Faculty of Advanced Sciences and Technology, Pharmaceutical Sciences Branch, Islamic Azad University (IAUPS), Tehran, Iran.

**Keywords:** Wound healing, Solid Lipid Nanoparticles, In-vivo, Semi solids

## Abstract

***Purpose:*** Wound healing is a natural biologic process, but the duration of it may take too long. Trying to shorten this process is one of the challenges for scientists. Many technologies were applied to achieve this goal as well as nanotechnology. In this study semi solid formulations containing curcumin and ampicillin solid lipid nanoparticles (SLNs) were prepared to evaluate as burn wound healing agent.

***Methods:*** Curcumin as an anti-inflammatory and anti-bacterial agent and ampicillin as an antibiotic were applied. In-vitro and in-vivo evaluations were carried out. Particle size, loading efficiency, release profile, morphology and anti-bacterial efficacy of desired nanoparticles were evaluated at first. Then the remaining of the antibacterial effect in semi solid preparations was studied. Animal studies for both toxicology using rabbits and skin burn model using rats were designed. Pathology studies after applying of formulations was done too.

***Results:*** Desired nanoparticles were spherical in shape and particle size in range of 112-121 nm, with low zeta potential. For increasing stability of particles they were freeze dried using cryoprotectant. Lyophilized particles show no significant size enlargement. Results showed that both ointment and gel preparations have reasonable anti-bacterial effects, both of them cause increasing in the rate of wound healing in comparison with placebos and control groups and none of the formulations showed acute toxicity.

***Conclusion:*** It seems that using nanotechnology could shorten wound healing process to reduce treatment costs and increase compliance of patients.

## Introduction

Wounds may occur different occasions in anyone’s life and wound healing is a biological process which involves four main phases including: hemostasis, inflammation, proliferation and maturation.^1,2^ Nanotechnology as a novel technology focuses on wound healing as well as many other medical complains. Ag, Au, Zn, Cu and NO nalso antibiotic containing nanoparticles are some of those which were used in wound healing.^3^ Most of mentioned nanoparticle show antibacterial activity and help to shortened wound healing process. Researchers demonstrated that nanofibers showed effectiveness in skin regeneration based on their structures which are similar to the extracellular matrix.^4^ Numerous innovative nanoscale products have emerged for wound healing that are currently under clinical investigation.^5^ Nash and Hugh demonstrated that topical ampicillin could help to reduce the incidence of infections after cancer operation of the large intestine also topical ampicillin showed a significant reduction in the frequency of infections after operation for cancer of the colon and rectum and side effects were not reported as well.^6^ Based on our knowledge there is no publication in which curcumin and ampicillin nanoparticles together apply via semi solid formulation for burn wound healing, in the present research we tried to evaluate the benefit of this rout of administration. Formulation of suitable dosage form which could be used for in-vivo studies was the main purpose of us. After achieving the optimum formulations, in-vitro, ex-vivo and in-vivo studies were carried out.

## Materials and Methods

### Preparation of SLNs

Curcumin and ampicillin solid lipid nanoparticles (SLNs) were prepared using high pressure homogenization method which was described by Varshosaz *et al* (2010). with justifications as needed.^7^ In brief active substances were dissolved/dispersed in water and tween 80 was added, in other side hot oily phase was prepared which contains cholesterol and ethanol/acetone (in volume ratio of 3/1), then oily phase was added to the watery phase under homogenization (13500 rpm). SLNs were prepared during homogenization and cooling of the mixture to the room temperature. Prepared SLNs were lyophilized using 5% mannitol as cryoprotectant, Varshosaz *et al.* (2012) Particle size, zeta potential, morphology, drug loading efficacy and antibacterial activities as well as drug release profile were investigated after freeze drying too.^8^

### Preparation of semisolid formulations

Ointments and gels were selected to be prepared for administering in animals.

#### Ointment preparation 

Petroleum (4.7 g) and Eucerin (4.3 g) were melted and cacao butter and olive oil, 0.2 and 0.6 g respectively, were added, 0.6 g glycerin was added to the mixture and under mixing and cooling condition to the room temperature, 255 and 192 mg lyophilized curcumin and ampicillin SLNs were added which were equal to 70 and 18 mg curcumin and ampicillin respectively. A soft ointment with an acceptable texture was made to apply.

#### Emulgel Preparation

0.8 g carbopol (971) added to distilled water (q.s 10 ml), then 2 and 3g olive oil and glycerin was added under mixer, after obtaining a soft gel texture, 255 and 192 mg of freeze dried curcumin and ampicillin SLNs was added and mixed to be homogenized.

Both formulations were evaluated for their viscosity using Brookfield DV III + Rheometer (USA).

#### Antimicrobial evaluation

Muller Hinton's agar plates were used the *in vitro* antimicrobial testing as recommended by clinical and Laboratory Standards Institute. Bacterial strains were sub cultured from frozen stocks. One of three loopful of 24 h old cultures from each test strains were used to prepare 0.5 McFarland standard suspensions. Each bacteria strain (*Escherichia coli, Staphylococcus aureus,* and *Pseudomonas aeruginosa*) were inoculated into Mueller-Hinton agar (MHA) plates to form a bacterial lawn. Then swabs soaked in different formulations and were stretched on the central inoculated MHA agar. The inoculated plates were incubated at 37°C and examined after 18-24 h. The zones of inhibition so obtained were measured and the results were compared. This study was performed in triplicate for each formulation.

#### Animal studies

#### Animal Housing and Maintenance for safety studies

Female rabbits were individually housed in separate quarters in solid bottom cages. Individual animals were identified by color coding, the animal number and group number also appeared on the outside of each cage to preclude mix-up. The animal room environment was controlled (targeted ranges: temperature 22°C to 25°C, relative humidity 30-20%) and monitored daily. The photo-cycle was 12 hours light and 12 hours dark .Upon arrival all animals were submitted to a general physical examination and all were found healthy and were admitted. Diet and water were offered ad libitum throughout the acclimatization and study periods. The cage cleaning schedule, air filtration and recirculation, health checks and facility maintenance were carried out in accordance with the IAUPS Standard Operating Procedures, and such activities were recorded in the animal room records.

#### Animal Selection/Randomization

The test population of animals was selected from newly arrived and the method of randomization was based upon the random selection of numbers generated from a set of numbers without replacement.

#### Skin Irritation and Corrosion Test

An acute skin irritation and corrosion study of the test article, Curcumin and ampicillin solid lipid nanoparticles (SLNs) was carried out before starting the burn model for topical application. The study was conducted according to OECD 404 protocol, Acute Dermal Irritation and Corrosion Test. The test substance was applied in a single dose to the skin of the first rabbit and untreated skin areas of the test animal serve as the control. The degree of irritation/corrosion was read and scored at specified intervals and was further described in order to provide a complete evaluation of the effects. The duration of the study was sufficient to evaluate the reversibility of the effects observed.

All rabbits received the test article by dermal application according to the test guideline Animals were observed individually at least once during the first 4 hours after dosing, periodically during the first 24, 48, 72 hours (with special attention given during the first 4 hours), and daily thereafter, for a total of 14 days, except where they need to be removed from the study and humanely killed for animal welfare reasons or are found dead. All observations were systematically recorded with individual records being maintained for each animal. Body weights were recorded before initiation of the treatment, and daily until the end of the study.

#### Animal’s manipulation for creating skin burn model

Adult male Wistar rats weighing 200–230 g were obtained from Animal house of Experimental Medicine Research Center of Tehran University of Medical Sciences. These animals were housed with ad libitum access to pellet food and water, under standard room temperature (22±2°C) on the 12hr light/dark cycles. The bedding materials for rats were replaced with new ones every day.

#### Experimental protocol for generation of burn skin model and burn healing

The animals were anaesthetized with an intraperitoneal injection of Ketamine (80 mg/kg) and xylazine HCL (10 mg/kg). The skin of dorsum was shaved and cleaned with 70% ethanol. An experimental skin burn model was induced on rats by the Edraki method.^9^ Second-degree burn wounds on the dorsum of the animals were exposed to ironic cylindrical devices with a surface of 2.5 cm2 for 10 seconds. The devices were heated in boiling water (98 ± 1 °C) at least 20 minutes before application to induce burn injury. Rats were randomly divided into either control or experimental groups. Control group did not receive any treatment. Experimental groups were subdivided into six groups as follows: Group 1 received topical ointment Group 2 received topical gel, Group 3 received blank of ointment formulation which contains all ingredients except desired nanoparticles, Group 4 received blank of gel formulation which contains all ingredients except desired nanoparticles, Group 5 and 6 received solution of curcumin and ampicillin and normal saline respectively. Each Rat treated with equal volume of topical formulation, once daily with regards to the assigned group and continued until 12 h before sample collection.

In order to collect samples for histopathology examination, the animals were euthanized at 7 and 14 days after inducing burn injury. These samples were preserved in 10% formalin for light microscopic study. Photography was also followed until the 21th day on the other wound of rats for wound surface measurements.

#### Histological assessments 

Tissue samples of burn wounds for histopathological analysis were immediately fixed in 10% formalin, and then the sections were embedded in paraffin. The section blocks of 5 μm were prepared and stained with hematoxylin and eosin (H&E) for histopathological examination by light microscopy. All skin tissues were examined in a blinded manner by a pathologist. Inflammation, collagen deposition, angiogenesis, granulation tissue formation and epithelialization were graded as 0-3 to each section.^10^ The burn wounds surface area, were photographed at the 0th, 5th, 11th, 14th, 18th and 21th days using a digital camera; then, the healing rate was evaluated by comparison of wound area at each control days with the wound area in the first day.^11^

## Results and Discussion

### Particle size and morphology evaluation of nanoparticles

**Figure 1:**
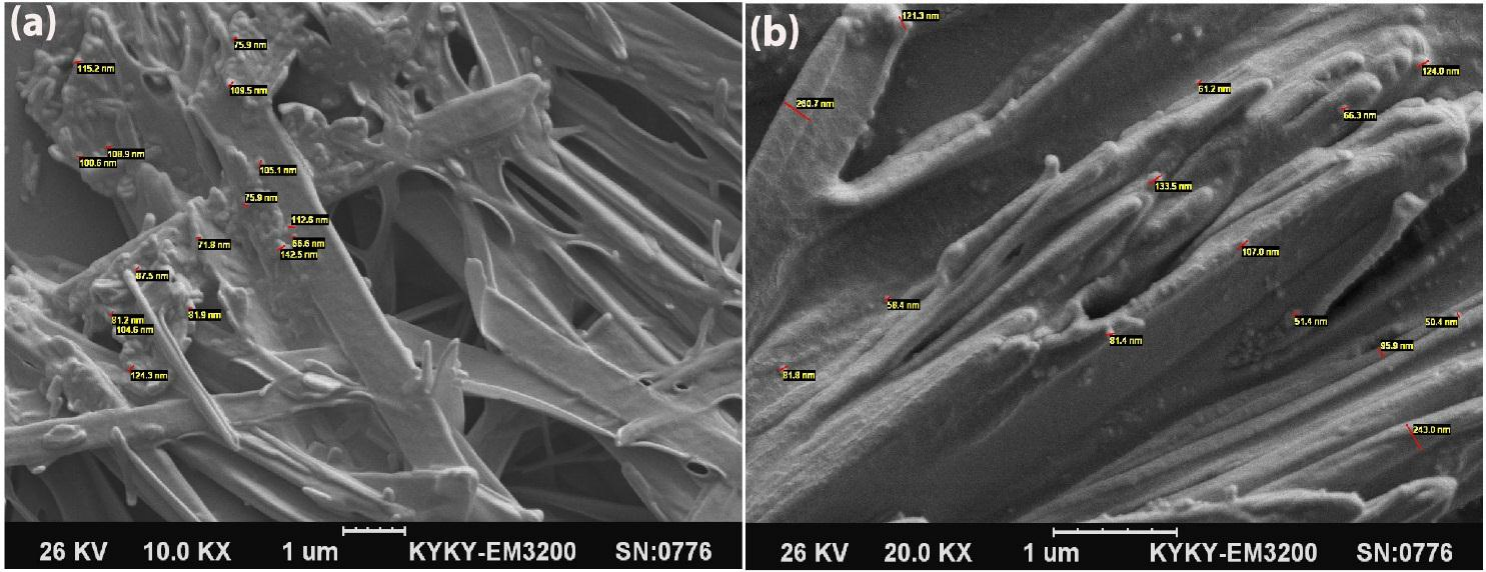
a: Curcumin Solid Lipid Nanoparticles photographs after freeze-drying(left). b: Ampicillin Solid Lipid Nanoparticles photographs after freeze-drying(right)

SEM photographs demonstrated that most of the particles are spherical even after freeze drying and non-significant size enlargement was seen after freeze drying. Figure 1a and 1b show SEM photographs of the desired nanoparticles.

### Antimicrobial efficacy of formulations

As antimicrobial efficacy enhancement using nanotechnology for both curcumin and ampicillin as well as synergic effect of using both nanoparticles containing curcumin and ampicillin were reported previously by Alihoseyni 2014, Jourghanian 2016 and Alihosseini 2016, this benefit of nanotechnology was evaluated in semisolid dosage forms in this project.^12-14^ Also previously Varaprasad et al (2011) studied on benefit of using curcumin with Ag hydrogel to enhance antibacterial efficacy. Their study results showed that curcumin could enhance anti bacterial efficacy of Ag nanoparticles.^15^

The “well diffusion test” was carried out using P.aueroginosa (ATCC 27853), S.aureus (ATTC 25923) and Ecoli (ATCC 25922), results show that antimicrobial effects of active ingredients which were loaded on SLNs remain in the formulation.

### Viscosity control

Viscosities of desired formulations were 1187 and 139.5 cps for ointment and gel formulation respectively.

### Animal studies

**Figure 2:**
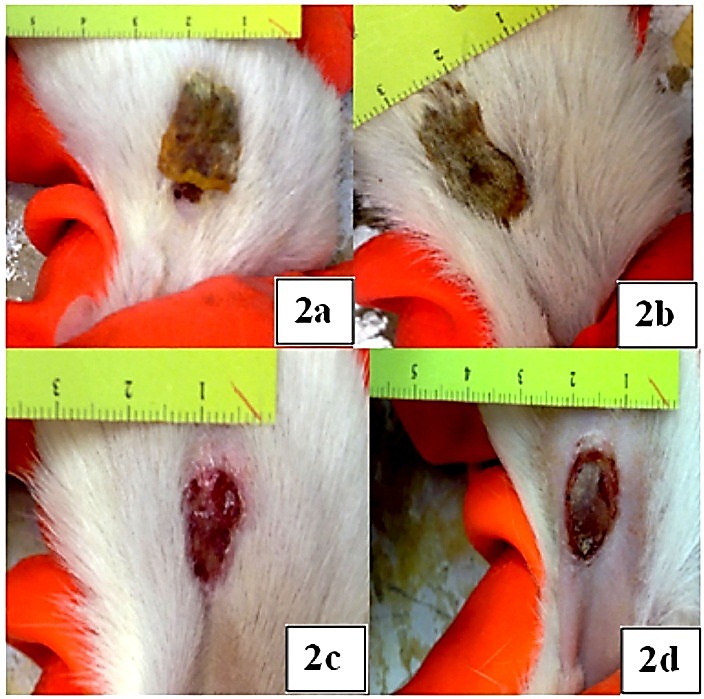
**a**: Group 1 skin condition after 14 days, **b**: Group 2 skin condition after 14 days. **c**: Skin condition of Group 3 after 14 days, **d**: Group 4 skin condition after 14 days

After applying formulations on the burned skin as described in methodology part, wound healing process studied for 14 days. Rate of healing based on wound appearance and diameter was evaluated in comparison with negative and positive controls as well as placeboes. Results show that by applying nano curcumin and nano ampicillin, rate of healing increases significantly. Figures 2(a, b, c, d) show photos of wound healing after using formulations in comparison with placeboes and normal saline. It is obvious that healing rate increases by applying nanoparticles. The pathology studies confirm the above results. Previously efficacy of curcumin on burn wound healing in comparison with silver sulfadiazine was studied by Mehrabani et al. (2017).^16^ They demonstrated that curcumin as an available and inexpensive herbal was shown be a suitable substitute in healing of burn wounds. Curcumin was encapsulated into a silane-hydrogel nanoparticle vehicle (curc-np) to overcome native curcumin's poor solubility and investigate its potential as a topical therapy for wound infection by Krausz et al.^17^ Curc-np demonstrated efficacy in vitro against methicillin-resistant Staphylococcus aureus and inhibited growth of this bacteria strain and enhanced wound healing in an in vivo burn wound model. These data suggest that curc-np may possess clinical utility as a novel topical antimicrobial and wound healing agent for infected burn wounds.^17^ Ahmadi et al demonstrated that using nanotechnology could enhance the rate of burn wound healing. The studied on Ag and tetracycline nanoparticles. Their findings support use of the AgNPs in combination with antibacterial medicine for the treatment of infectious skin wounds.^18^ present study is correlated with the Krausz and Ahmadi's researches results.

### Pathology studies

**Figure 3:**
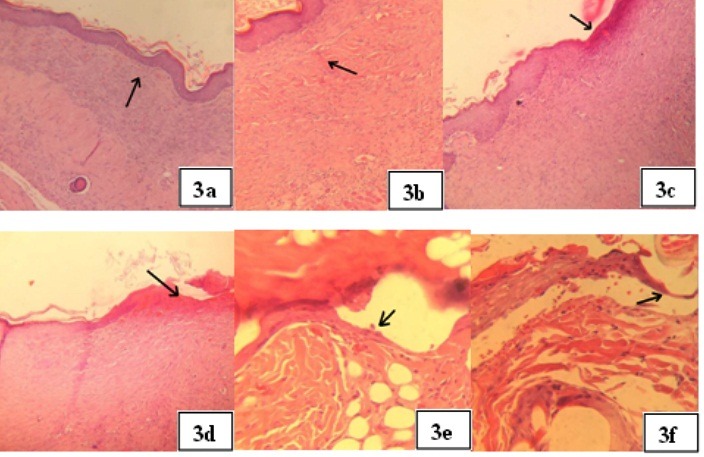
**a**: belongs to Group 1 after 14 days applying ointment formulation and shows showed tissue healing with complete and mature re-epithelialization, fibroblast proliferation, and collagen fiber. (mag:200×)**. b**: belongs to Group 2 after 14 days applying gel formulation and shows complete and mature epithelization. There was severe fibroplasias (arrow) with high level of collagen fiber. (mag:400×)**. c**: belongs to Group 3 after 14 days applying placebo of ointment formulation and shows incomplete epithelization(arrow) with severe fibrosis and moderate inflammatory cells infiltration. (mag:200×).**d**: belongs to Group 4 after 14 days applying placebo of gel formulation and shows partial epithelization(arrow).there was moderate fibrosis in wound area. (mag:200×)**. e**: is belonging to Group 5 In which Curcumin and ampicillin solution was applied, after 14 days complete, but thin epithelization (mag:400×) was observed. **f**: is belonging to Group 6 in which Normal saline was applied and photograph was taken after 14 days. Revealed partial epithelization.(mag:400×) is obvious

Pathology studies confirm that nanotechnology enhances wound healing process. Figure 3(a, b, c, d, e and f) show the results.

As wound healing is a natural but time consuming process, in control groups healing observed but slower than when nanoparticles were applied.

### Skin Irritation and Corrosion Test 

All animals were examined for signs of erythema and edema, and the responses scored at 60 minutes, and then at 24, 48 and 72 hours after patch removal. For the initial test in one animal, the test site was also examined immediately after the patch had been removed. Dermal reactions were graded and recorded according to the grades in Table 1 as below. If there was damage to skin which could not be identified as irritation or corrosion at 72 hours, observations were continued until day 14 to determine the reversibility of the effects. In addition to the observation of irritation, all local toxic effects, such as defeating of the skin, and any systemic adverse effects (e.g., effects on clinical signs of toxicity and body weight), were fully described and recorded.

Observations of the first examined rabbit were described in Table 1. The proposed SLNs exposed areas didn’t show any sign of toxicity after first 4 hrs from dermal application. In long term continuous dermal exposure, very slight erythema and dryness were observed but these mild signs of toxicity were recovered to normal appearance after the removal of the patch in case areas. The mean total score was 4 in treated rabbits therefore the product showed very slight irritant effect without any corrosive response in long term (>24hrs) exposure. After the recovery period all animals had normal appearance and hair growth in treated areas.

**Table 1:** Mean (SD) skin reactions of 3 rabbits 1 after 3min,1hrs,4 hrs,24 hrs,48 hrs and 72 hrs dermal exposure to proposed Solid Lipid Nanoparticles

**Erythema and Eschar Formation**	**3min**	**1hr**	**4hr**	**24hr**	**48hr**	**72hr**	**Edema**	**3min**	**1hr**	**4hr**	**24hr**	**48hr**	**72hr**
**No erythema**	0	0	0	0	0	0	**No edema**	0	0	0	0	0	0
**Very slight erythema (barely perceptible)**	0	0	0	1	1	1	**Very slight edema**	0	0	0	0	0	0
**Well defined erythema**	0	0	0	0	0	0	**Slight edema**	0	0	0	0	0	0
**Moderate to severe erythema**	0	0	0	0	0	0	**Moderate oedema**	0	0	0	0	0	0
**Severe erythema**	0	0	0	0	0	0	**Severe edema**	0	0	0	0	0	0
**Skin dryness**	0	0	0	0	1	0	**Weight of Animals :3865±108**						
**Total score:4**	0	0	0	1	2	1							

### Bodyweight 

Individual weights of animals were determined shortly before the test substance was administered and for more two weeks thereafter. Weight changes were calculated and recorded. At the end of the test surviving animals were weighed. No significant weight change was detected in this test.

Based on the foregoing results, the SLNs samples didn’t show any sign irritant effects even after long term (>72 hrs) in treated animals when compared with control areas in Albino Rabbit.

## Conclusion

In this project, synergic antibacterial effect of ampicillin and curcumin SLNs which was demonstrated previously, was applied to treat burn wound. Two semisolid preparations were designed to use on burned skin. The combination of curcumin and ampicillin nanoparticles was evaluated as wound dressing materials in animal wound models. Application of the dressings showed significant improvement in wound healing. These two nanoparticles have synergistic antibacterial effect together. It seems that using nanotechnology could help to increase rate of wound healing.

## Acknowledgments

Research reported in this publication was supported by Elite Researcher Grant Committee under award number [‏943784] from the National Institutes for Medical Research Development (NIMAD), Tehran, Iran.

Authors would like to appreciate Laboratory of veterinary pathology with managing of Dr Hasti Azarabad for pathology studies. Authors also would like to appreciate kind assistance of Mrs. Hedieh Ghaffari for English polishing of the manuscript.

## Ethical Issues

The experimental performed during this study were approved by Animal Ethics Committee of Tehran University of Medical Sciences as well as Animal Care Guidelines Published by the Department of Pharmacology, School of Medicine, Tehran University of Medical Sciences.

## Conflict of Interest

There is no conflict of interest to declare.
