# Erratum to "Nanotechnology in Wound Healing; Semisolid Dosage Forms Containing Curcumin-Ampicillin Solid Lipid Nanoparticles, *in Vitro*, *Ex Vivo* and *in Vivo* Characteristics"

**DOI:** 10.34172/apb.2021.088

**Published:** 2020-11-07

**Authors:** Solmaz Ghaffari, Faezeh Alihosseini, Seyed Mahdi Rezayat Sorkhabadi, Sepideh Arbabi Bidgoli, Seyyedeh Elaheh Mousavi, Setareh Haghighat, Ahoo Afshar Nasab, Nooshin Kianvash

**Affiliations:** ^1^Pharmaceutical Sciences Research Center, Pharmaceutical Sciences Branch, Islamic Azad University (IAUPS), Tehran, Iran.; ^2^Young Researchers and Elite Club, Pharmaceutical Sciences Branch, Islamic Azad University (IAUPS), Tehran, Iran.; ^3^Department of Medical Nanotechnology, School of Advanced Sciences and Technology in Medicine, Tehran University of Medical Sciences (TUMS), Tehran, Iran.; ^4^Department of Pharmacology and Toxicology, Pharmaceutical Sciences Branch, Islamic Azad University, (IAUPS), Tehran, Iran.; ^5^Department of Toxicology, Pharmaceutical Sciences Branch, Islamic Azad University (IAUPS), Tehran, Iran.; ^6^Department of Pharmacology, School of Medicine, Tehran University of Medical Sciences, Tehran, Iran.; ^7^Department of Microbiology, Faculty of Advanced Sciences and Technology, Pharmaceutical Sciences Branch, Islamic Azad University (IAUPS), Tehran, Iran.

This erratum is issued to amend the version of record of “Nanotechnology in Wound Healing; Semisolid Dosage Forms Containing Curcumin-Ampicillin Solid Lipid Nanoparticles, *in-Vitro, Ex-Vivo* and *in-Vivo* Characteristics” published in “Advanced Pharmaceutical Bulletin”, 2018, 8(3), 395-400. doi: 10.15171/apb.2018.046

The journal’s editorial staff introduced a number of textual and typographical changes to the earlier version of the published article text. These changes were approved by the authors, and resulted in an improved readability level of the text as well as some shifts in the scientific meaning of the submitted work.

By issuing the correction, “*Advanced Pharmaceutical Bulletin*” hopes that the readability and the original meaning and emphasis will be restored, and so allow readers the full benefit of this research. The journal has taken steps to ensure that measures are in place to avoid publishing an incorrect version of an article in the future. The “*Advanced Pharmaceutical Bulletin*” apologizes for these errors and any consequent inconvenience to the readers. The article was corrected online.

## References

[1] Ghaffari S, Alihosseini F, Rezayat Sorkhabadi SM, Arbabi Bidgoli S, Mousavi SE, Haghighat S (2018). Nanotechnology in wound healing; semisolid dosage forms containing curcumin-ampicillin solid lipid nanoparticles, in-vitro, ex-vivo and in-vivo characteristics. Adv Pharm Bull.

